# Special Issue on “Disseminated Intravascular Coagulation: Current Understanding and Future Perspectives”

**DOI:** 10.3390/jcm11123315

**Published:** 2022-06-09

**Authors:** Kazuma Yamakawa

**Affiliations:** Department of Emergency and Critical Care Medicine, Osaka Medical and Pharmaceutical University, Takatsuki 569-8686, Japan; kazuma.yamakawa@ompu.ac.jp

Disseminated intravascular coagulation (DIC) is characterized by the systemic activation of blood coagulation that generates and deposits fibrin that causes microvascular thrombi to develop in various organs, which contributes to multiple organ dysfunction [1,2]. Simultaneous neutrophil activation and endothelial injury associated with the disruption of the glycocalyx have also been observed in these patients [3]. Although several recent advances have been achieved in understanding the pathophysiology of DIC, the clinical significance of diagnoses and therapeutic interventions targeting DIC continues to remain uncertain. Therefore, understanding the pathophysiology of DIC and realizing novel therapeutic strategies are vitally important for the management of patients in critically ill settings.

In January 2020, our Special Issue titled “Disseminated Intravascular Coagulation: Current Understanding and Future Perspectives” was launched in the *Journal of Clinical Medicine*. Many articles were published concerning different topics in DIC, including novel studies on promising diagnostic techniques and the treatment of the syndrome. We introduce these papers below, and encourage frontier papers on novel findings for the subsequent series.

## 1. Etiology and Pathophysiology of DIC

Blood coagulation disorders commonly occur in patients suffering from severe coronavirus disease 2019 (COVID-19), but the evidence on differentiating the pattern of hemostatic abnormalities from those of typical sepsis-induced DIC remains limited. In a single-center, retrospective, observational study that compared hemostatic biomarkers between 24 patients with severe COVID-19 and 200 patients with non-COVID-19 sepsis, Umemura et al. revealed the specific pattern of coagulopathy induced by COVID-19 [4]. Although the platelet count, antithrombin activity, and prothrombin time in the COVID-19 group remained almost within normal range, fibrin/fibrinogen degradation products and D-dimer were significantly higher in the COVID-19 group versus the non-COVID-19 sepsis group. Contrastingly, moderately high levels of the thrombin–antithrombin complex and plasmin–alpha2–plasmin inhibitor complex were present in the COVID-19 patients, but their levels of plasminogen activator inhibitor-1 were normal. Consequently, these authors concluded that a quite different hematological phenotype is present in COVID-19-induced coagulopathy compared with that seen in typical sepsis-induced DIC, in which local thrombus formation might be promoted in severe COVID-19. Now, even though a clear treatment option using anticoagulants is available to patients with moderate-to-severe COVID-19 [5,6,7], a further clarification of the mechanisms of the coagulation abnormality is still warranted.

Persistent inflammation, immunosuppression, and catabolism syndrome (PIICS) is a recently discovered phenomenon, in which prolonged inflammation associated with immunodeficiency and catabolism occurs after acute-phase treatment. Nakamura et al. first reported an independent association between DIC and subsequent PIICS development in critically ill patients [8]. In their retrospective analysis of 5397 patients (488 with PIICS, 416 with early death, and 4493 without PIICS), these authors showed a significant association between DIC diagnosed according to the International Society on Thrombosis and Haemostasis (ISTH) over the DIC scoring system and mortality, the Barthel index (which assess activities of daily living) at discharge, and the development of PIICS. Importantly, the presence of DIC at admission in the surviving patients was found to be an independent risk factor for PIICS.

Purrucker et al. used a large single-center database in Germany to analyze the etiology of ischemic strokes in 341 patients, despite their receiving anticoagulation with vitamin K antagonists (VKA) (*n* = 127) or non-VKA oral anticoagulants (NOAC) (*n* = 214) [9]. They found an overall increase in the rate of oral anticoagulant-associated stroke occurring per year. Among the patients with a sufficient diagnostic work-up, 95.3% of them were found to have at least one other potential, unclear, or unlikely cause of stroke that was not cardiac-based. Further, medication errors were more prevalent in the VKA versus NOAC group. This paper revealed two clinically important findings: (1) despite atrial fibrillation, risk factors for stroke remained highly prevalent, and (2) medication errors remain frequent, although less so for NOAC than for VKA.

## 2. Early Diagnosis of DIC

The development of novel techniques to improve the early diagnosis and treatment of DIC are of extreme importance in this field of research. By using machine-learning techniques with a nationwide registry in Japan, Hasegawa et al. attempted to establish novel models to predict the progression of coagulopathy [10]. They calculated the delta-DIC score as the ISTH DIC score on day 3 minus that on day 1, and compared the predictive accuracies of three common machine-learning methods—random forests, support vector machines, and neural networks—with those of conventional methods. The resulting predictive accuracies for the development of late-onset DIC ranged from 59.8% to 67.0%. As these predictive accuracies were clinically unsatisfactory, further research to develop other model fits for clinical application is needed.

Kim et al. investigated the role of thromboelastography (TEG) as an early predictor of DIC in patients with septic shock [11]. They included 889 patients (158 with DIC, 731 without DIC) who underwent TEG in the emergency department. The highest discriminating power for DIC was the TEG value of maximal amplitude, with an area under the ROC curve of 0.814. Further, a multivariable analysis showed maximal amplitude to be an independent predictor of DIC, and the authors, thus, concluded that this TEG measurement value could be valuable for the early prediction of DIC in patients with septic shock.

## 3. Therapeutic Strategy against DIC

Nafamostat mesylate (NM) is a synthetic serine protease inhibitor that can be used both as an anticoagulant during blood purification in critically ill patients and as a treatment for DIC. Although NM can reduce the risk of bleeding during blood purification procedures when compared with heparin, its effect on mortality remains unknown. Kamijo et al. used a nationwide Japanese registry of patients with sepsis treated with blood purification to evaluate the association between NM administration and in-hospital mortality [12]. They compared 268 pairs of propensity score-matched patients receiving either NM or conventional therapy. The patients receiving NM showed significantly lower rates of hospital and intensive care unit mortality than those receiving the conventional therapy, and these authors noted that NM administration might improve survival outcomes of patients with sepsis who require blood purification.

My group previously performed an umbrella review (review of systematic reviews or meta-analyses) to provide an overview of the existing systematic reviews of randomized controlled trials of anticoagulant therapy in sepsis [13]. Our analysis of 19 systematic reviews showed that in poorly characterized patient groups, no beneficial effect was observed in overall sepsis; however, in more specific patient groups with sepsis-induced DIC or sepsis with coagulopathy, a beneficial effect was observed. Several reports [14,15,16,17,18] have repeatedly shown how import adequate patient selection is in the therapeutic strategy against DIC. Nevertheless, as no DIC treatment has been well-established by robust evidence, further investigation is warranted.

Lastly, given the enormous success of the release of the first volume of this Special Issue, I believe that it is time to move forward to a second volume to collect additional insights into DIC. In this subsequent Special Issue of the *Journal of Clinical Medicine*, we aim to discuss the etiology, pathophysiology, clinical manifestations, diagnostics, and optimal management involved in treating critically ill patients with DIC. As we are very keen to attract a global audience, we welcome any contributions on this subject from around the world.
